# Does IL-17 Respond to the Disordered Lung Microbiome and Contribute to the Neutrophilic Phenotype in Asthma?

**DOI:** 10.1155/2016/6470364

**Published:** 2016-01-28

**Authors:** Xu Yang, Yunqiu Jiang, Changzheng Wang

**Affiliations:** Institute of Respiratory Diseases, Xinqiao Hospital, Third Military Medical University, Chongqing 400037, China

## Abstract

Th17/IL-17 plays an important role in host defense and hyperimmune responses against pathogenic bacteria accompanied by the recruitment of neutrophils. Th17-associated immune response is also involved in the pathogenesis of asthma, which is known as a noninfectious allergic airway disease and has been shown to be heterogeneous. Th17-associated inflammation usually contributes to the neutrophilic phenotype, which is often characterized by greater severity, airflow obstruction, and steroid resistance. Concurrently, advanced culture-independent molecular techniques have increased our understanding of the lung microbiome and demonstrated that disorders of the lung microbiome, including changes of the total burden, diversity, and community composition, may contribute to severe, treatment-resistant neutrophilic asthma, although the precise mechanism is still unclear. Because Th17/IL-17 plays a role in bacteria-mediated immune responses and is involved in neutrophilic asthma, there may be a link between them. We review the effects of Th17/IL-17 on bacteria and asthma, showing the possibility that Th17/IL-17 may be a key player in neutrophilic asthma which may be characterized as severe or treatment-resistant by responding to the disordered lung microbiome.

## 1. Introduction

Th17 cells are known as a distinct lineage of CD4^+^ T cells, which are promoted by antigen-presenting cells (APCs) through IL-1*β* (in humans)/TGF-*β*1 (in mice), IL-6, and IL-23 [1–4]. Th17 cells are one of the main sources of IL-17 (all referring to IL-17A in this review), which functions by recruiting neutrophils [5]. Neutrophils play an important role in preventing bacterial dissemination and increasing the clearance of pathogens [6] and promote inflammatory responses and mediate tissue injury [7, 8].

In addition to bacteria-mediated diseases, Th17/IL-17 has also been found to be elevated in asthma with levels that are positively correlated with disease severity [9–12]. Because it functions by recruiting neutrophils, Th17-associated inflammation contributes more towards neutrophilic asthma [13], one of the inflammatory phenotypes of asthma, which is often characterized by worse control levels, a greater need for inhaled corticosteroids, and treatment resistance compared to other phenotypes [14, 15].

The development of molecular technology has largely expanded our understanding of the lung microbiome. The lower airway is no longer thought of as sterile. Disorders of bacterial microbiota have been found in the airways of asthmatics [16], and the observed community composition has been shown to differ among different features and severity of asthma [17].

Because the disordered lung microbiome is involved in the pathogenesis of neutrophilic asthma and Th17/IL-17 plays a role in neutrophil recruitment in both bacteria-mediated diseases and asthma, there may be a link between Th17/IL-17-associated immune responses to some specific bacterial microbiota and the pathogenesis of severe or steroid-resistant neutrophilic asthma. In this review, we describe the possibility of the link based on recent discoveries.

## 2. IL-17 and Bacteria-Mediated Immune Responses

IL-17 mediates innate and acquired immunity to certain strains of bacterial infection to aid in host defense. In mice, IL-17 has been found to increase the clearance of bacteria by recruiting macrophages and neutrophils in the context of* Streptococcus pneumoniae* infection [18]. This protection was defective in both IL-17 receptor knockout (KO) and neutrophil-depleted mice [19]. In acute* Pseudomonas aeruginosa* infection, Th17 cells and IL-17 levels also increased and induced the recruitment of neutrophils in the early period, which played protective roles [20]. In* Klebsiella pneumoniae* infection, IL-17 supported protection through the induction of granulocyte colony-stimulating factor (G-CSF) and neutrophil recruitment [21]. For intracellular pathogens, IL-17 also mediates bacterial killing and host responses by regulating IL-12-Th1 cell immunity [22]. In* Mycobacterium tuberculosis* resistance, IL-17 contributed to granuloma formation and CXCL13 expression [23], and, in IL-17 KO mice, granuloma formation was found to be impaired after infection with* Mycobacterium bovis* Bacille Calmette-Guérin (BCG) [24]. Although our understanding of IL-17 in human host defense remains limited, studies with the aforementioned animal models have provided evidence for the role of IL-17 in mammalian immune responses against pathogens and the importance of neutrophil recruitment and activation is involved in the mechanisms.

Moreover, IL-17 also plays a complex role in bacteria-mediated hyperimmune response. Hypersensitivity pneumonitis (HP) is an inflammatory disease that can progress to lung fibrosis. Simonian et al. demonstrated that, in a* Bacillus subtilis*-induced HP model, IL-17 expressed by *γδ* T cells play a role in bacterial clearance and downregulate inflammatory responses and lung fibrosis [25, 26], whereas in another* Saccharopolyspora rectivirgula*-induced HP model, IL-17 expressed by CD4^+^ T cells, which acted as a proinflammatory cytokine, produced an unregulated inflammatory and fibrotic response and promoted rather than protect against hypersensitivity pneumonitis and lung fibrosis [7]. The two opposing effects suggest that the role of IL-17 in the pathogenesis of infectious and inflammatory diseases may differ depending on the microorganisms used in mouse models and cells that produce it.

## 3. IL-17 and Asthma

In addition to bacteria-mediated diseases, Th17/IL-17 has also been known to be involved in airway diseases, such as asthma [11], in which bacterial microbiota was traditionally thought to not play a role.

Asthma is a chronic inflammatory disease, which has been shown to be heterogeneous [27]. Indeed, less than 50% cases of asthma are attributed to atopy and present as typical Th2 inflammation [28, 29]. According to the proportions of neutrophil and eosinophil in induced sputum, asthma is categorized into 4 inflammatory subtypes: neutrophilic asthma, eosinophilic asthma, mixed granulocytic asthma, and paucigranulocytic asthma [30]. Whereas eosinophilic asthma is usually driven by Th2-associated inflammation, neutrophilic asthma correlates more strongly with Th17-associated inflammation [31].

IL-17 mRNA and protein levels have been found to be elevated in the lung tissue, induced sputum, and serum of asthmatics and to correlate with increased disease severity [9–12]. As IL-17 plays a role in recruiting neutrophils into the airways [11, 31, 32], some severe asthma patients exhibit airway neutrophilic inflammation, which is induced by Th17 cells and linked to elevated IL-17 [13, 14, 33]. These patients often appear to have difficulty in gaining improvements in symptoms and forced expiratory volume in one second (FEV1) [15]. This difficulty may partly be due to the resistance of Th17 cells to corticosteroid treatments [34]. Moreover, IL-17 induces human bronchial and murine epithelial cells to express Muc5ac and Muc5b [35], contributing to goblet cell hyperplasia, airway remodeling, steroid resistance, and the pathogenesis of severe or refractory asthma [36–38].

The precise mechanisms of Th17-associated neutrophilic inflammation and how it contributes to worsened clinical outcomes remain unclear. Because IL-17 plays an important role in host defense and hyperimmune responses against bacteria, are there any scenarios in which bacteria may be involved in the pathogenesis of Th17 inflammation in neutrophilic asthma?

## 4. Lung Microbiome

The lower airway is not sterile. Advanced culture-independent molecular techniques, including qPCR and 16S rDNA sequencing, have challenged our understanding of the lung microbiome [39].

Charlson et al. analyzed the bacterial abundance and composition throughout the respiratory tract by sampling at multiple sites in six healthy individuals, showing that bacteria were resident in both upper and lower airways of healthy people [40]. Compared with the upper airways, the lung microbiome displayed similar community composition but less biomass, suggesting possible aspiration of colonizing microorganisms from the upper airways [40]. This aspiration occurs commonly in healthy people [41]. It increases the risk of smoking-related lung diseases, for smoking significantly alters the species in the oro-/nasopharynx, although little direct influences have been found on the lung microbiome [42, 43]. When significant quantities of disordered supraglottic bacteria aspirate into the lungs, the normal relative abundance of the lung microbiota will change and subclinical lung inflammation increases [44]. This phenomenon may in part play a role in the development of COPD triggered by cigarette smoking.

Another study investigated the respiratory tract microbiome in 64 healthy individuals, and some specific bacteria, including Enterobacteriaceae,* Haemophilus*,* Methylobacterium*, and* Ralstonia*, were found to have much higher abundance in the lungs, indicating that not all bacteria in the lungs were derived from the oral cavity [43].

Alterations of the lung microbiome also contribute to pulmonary inflammation and participate in the pathogenesis of various airway inflammatory diseases. Rutebemberwa et al. showed that* Novosphingobium* spp. presented and played a role in more severe COPD [45]. In non-cystic fibrosis bronchiectasis, the loss of bacterial diversity in the lower airway is correlated with decreased FEV1 [46]. A significant association between idiopathic pulmonary fibrosis (IPF) disease progression and the relative abundance of* Streptococcus* and* Staphylococcus* genera has also been reported [47]. Moreover, exacerbations without acute infections during the aforementioned diseases have also been found to associate with increased bacterial burdens and decreased community diversity [48].

## 5. Lung Microbiome in Asthma

Although asthma is usually known as a noninfectious allergic disease, views challenging current concepts have emerged, which indicate an association between disordered bacterial microbiota and pathogenesis of asthma. Wood et al. reported that several potentially pathogenic bacteria with significant quantities were cultured from the sputum in 15% (17/115) of patients with stable asthma with increases of sputum total cell counts, the proportion and number of neutrophils, and IL-8 levels, suggesting the presence of lung microbiota and its effects on immunity in asthma [49]. With culture-independent 16S rDNA sequencing, Hilty et al. [50] found more bacterial microbiota in the airways of patients with asthma or COPD compared to healthy controls. All of the subjects enrolled were free of clinical infections and antibiotics in this study. The results showed significant increases in pathogenic Proteobacteria, particularly* Haemophilus* spp., in asthmatics and increases in Bacteroidetes, particularly* Prevotella* spp., in controls. However, glucocorticosteroids, the main treatment for asthma, promote the persistent colonization of* Haemophilus influenzae* in a mouse model of infection [51]. Thus, whether asthma or steroid treatment should be responsible for the disorder of the lung microbiome remains unclear. Marri et al. [16] collected the induced sputum samples from mild active asthmatics and nonasthmatics. Compared with those of nonasthmatics, sputum samples from patients contained higher proportions of Proteobacteria, which was consistent with the findings of Hilty et al., and possible lower proportions of Firmicutes and Actinobacteria. Because 80% of patients enrolled in this study were not using corticosteroids, the changes in the relative abundance of bacterial species should be attributed to the disease itself rather than the treatments. Another study which investigated the combined effects of colonization and allergic airway disease also showed a declined clearance rate of* Haemophilus influenzae* in OVA-sensitized mice, indicating the possibility that allergic airway diseases, such as asthma, contribute to chronic bacterial colonization and alterations of the lung microbiome [52]. Steroid treatment further promotes dysbiosis and exerts synergistic effects.

The lung microbiome also differs in patients with different asthma control levels and disease severity. In suboptimally controlled asthmatics, the burden and diversity of bronchial microbiota have been reported to be much higher, and an association between bacterial composition and degree of airway hyperresponsiveness was also investigated [53]. Moreover, compared to mild-moderate asthmatics, patients with severe asthma were significantly enriched in taxa belonging to Actinobacteria and had less abundant levels of several different families of Proteobacteria [17].

In contrast, however, one study compared the pathogenic microorganisms of the lungs, including bacteria, virus, and fungi, in predominantly allergic asthma, nonallergic asthma, mixed asthma, and unspecified asthma patients according to the latest edition of the ICD (J45.0–J45.9) from 2011 to 2012 with no limitations on the patients' state of exacerbation using methods including Gram staining, culture, and PCR and found no significant differences among the 4 groups [54]. Therefore, associations between asthma and microorganisms have not been firmly established.

The less sensitive methods used in this study may have been responsible for the negative result, but the inappropriate subject grouping may have had even more of an effect. In this study, subjects were grouped by whether they were allergic or not, but no direct associations between bacterial products and allergy have been found yet. In contrast, the dose-dependent effect of LPS on immune responses has been determined. In a mouse model using OVA challenges, very low doses of LPS induced Th2-dominant inflammation, whereas Th17-related inflammation was induced and promoted by increased doses of LPS. When the dose of LPS continued to increase and reached levels much higher than those found in the environment, large numbers of Treg cells accumulated [55]. Therefore, the major effect of the lung microbiome on asthma is likely to shape immunity and subsequently influence clinical manifestations and disease severity. Because of the importance of immune responses in the pathogenesis of asthma with disordered lung microbiomes, the fact that enrolled subjects comprised both attack and attack-free patients would aggravate the immune heterogeneity among the groups and veil any potential differences.

## 6. Bacterial Disorder in Asthma and Involvement of IL-17

The greater share of evidence indicates that disorders of the bacterial microbiota play a role in asthma development, corticosteroid response, and severity by regulating immunity with IL-17 involved in this regulation.

Birth cohort studies in Copenhagen revealed that neonates with bacterial colonization, especially* S*.* pneumoniae*,* H*.* influenzae*, or* Moraxella catarrhalis*, had a higher risk for asthma in their early lives [56]. These pathogenic bacteria may promote different types of immune responses. Følsgaard et al. reported that colonization by* M*.* catarrhalis* and* H*.* influenzae* induced a mixed Th1/Th2/Th17 response, whereas colonization by* S*.* aureus* induced a Th17 response with elevated IL-17 levels [57]. The colonization might be associated with acute wheezing episodes [58], but even if asymptomatic in infancy, children diagnosed with asthma at the age of 7 years still exhibited excessive immune responses with the aberrant production of IL-5, IL-13, IL-17, and IL-10 by their PBMCs when treated with those pathogens that had colonized them during their infancy [59]. These studies indicate that some specific pathogenic bacterial genera induced Th17-associated immune responses and may be responsible for increased risk of asthma. However, some other studies support the alternative hypothesis that exposure to diverse microbes in early life helps to protect against atopic diseases. Gollwitzer et al. found that the shifts of lung microbiome from predominant phyla of Gammaproteobacteria and Firmicutes towards Bacteroidetes may be a key factor that induces Treg cells via PD-L1, promoting the tolerance to allergen during the maturation of the neonatal immune system and decreasing the responsiveness to allergen in adulthood [60]. Therefore, the actual effect on asthma development may be determined by the complex interactions among the burden, genera, microbe diversity, the types and routes of exposure, and host immunity [61].

A clinical trial investigating 42 preschool children with severe persistent wheezing and without symptoms of acute respiratory infections revealed that 81% of all subjects had neutrophilic inflammation and elevated bacteria counts presented in 59% among them. After treatment with antibiotics, 92% of the subjects obtained significant improvements, suggesting that bacterial colonization was responsible for the increased severity of symptoms and elevation of neutrophils [62]. To directly describe the inflammatory phenotype-specific alterations to the airway microbiome in asthma, Simpson et al. [63] grouped poorly controlled asthmatics according to their inflammatory phenotypes and analyzed their sputum microbiome, showing that neutrophilic asthma had expanded* Haemophilus influenzae* with reduced bacterial diversity and species richness, whereas eosinophilic asthma had greater percentages of* Tropheryma whipplei*. This study directly and strongly confirmed the disorder of lung microbiome in neutrophilic asthma.

As discussed previously, neutrophilic asthma is often characterized by a positive correlation with disease severity and steroid-resistant airway inflammation. Th17/IL-17-associated immune responses exhibit the same characteristics accompanied by recruiting neutrophils. Thus, Th17/IL-17 immune response likely acts as a bridge between the lung microbiome and neutrophilic asthma.

In steroid-resistant asthma, the dominance of* Moraxella catarrhalis* or* Haemophilus* or* Streptococcus* genera in the induced sputum is associated with a longer asthma disease course, worse postFEV1%predict (68.0% in average), and higher proportions and numbers of sputum neutrophils, which are also correlated with the total bacterial burden [64]. Another study that compared the lung microbiome composition in patients with corticosteroid-resistant (CR) and corticosteroid-sensitive (CS) asthma also showed the differences at genus level and that* Haemophilus* spp. are the most responsible for corticosteroid resistance [65]. In a murine model of allergic airway disease,* Haemophilus influenzae* colonization rather than active infection successfully induced steroid-resistant neutrophilic inflammation that was mediated by IL-17, suggesting the involvement of IL-17 in refractory neutrophilic asthma associated with disordered lung microbiome [52, 66].

In severe asthma, compared to mild-moderate asthma, taxa most belonging to Actinobacteria were found to be significantly enriched, whereas several different families in Proteobacteria were much less abundant [17]. More importantly, these researchers also analyzed the association of airway bacterial microbiota (sampled by bronchial brushings) and host epithelial gene expression, showing that several families in Proteobacteria positively correlated with Th17-associated gene expression [17]. However, interestingly, these families were different from those that belonged to Proteobacteria but correlated with FKBP5, a marker of steroid resistance. Proteobacteria levels were also negatively correlated with biopsy eosinophil numbers. Therefore, these results suggest that the increase of specific airway bacterial microbiota, such as some families in Proteobacteria, leads to Th17-associated inflammation and contributes to noneosinophilic asthma or neutrophilic asthma through the recruitment of neutrophils by IL-17. And this was independent of steroids effects. The involvement of IL-17 in neutrophilic asthma with a disordered microbiome has also been demonstrated in animal models. In a mouse model,* Streptococcus pneumonia* infection in infancy increased the risk of adulthood allergic airway diseases, with elevated Th17/IL-17 levels and accumulation of neutrophils, whereas the neutralization of IL-17 by monoclonal antibody significantly reduced neutrophil recruitment, alleviated airway inflammation, and decreased airway hyperresponsiveness, indicating an essential role of IL-17 in the pathogenesis of neutrophilic asthma [67].

## 7. Conclusion

The bronchial tree is not sterile. Lung microbiome disorders are involved in many chronic airway diseases, such as asthma. When some specific potentially pathogenic bacteria colonizing in the airways increase, the total burden and/or the community composition of the lung microbiome may change, which may subsequently induce Th17-associated inflammation. Large amounts of IL-17 are released and neutrophils are recruited into the airways, contributing to neutrophilic inflammation and promoting host defense or bacteria-mediated immune responses. Moreover, these responses are likely involved in the pathogenesis of severe, steroid-resistant neutrophilic asthma, which has been shown to be associated with the disordered lung microbiome. However, more studies on the precise mechanisms of how Th17/IL-17-associated immunity responds to the lung bacterial microbiota and determines the inflammatory and clinical phenotypes of asthma should be performed to help further understand the complex pathogenesis of asthma and generate more therapeutic options.
